# The implementation of a “safety officer” program: an innovative approach to improve infection prevention and control practices in Ethiopia

**DOI:** 10.3389/fpubh.2024.1448655

**Published:** 2024-10-09

**Authors:** Solomon Abebe Woldeamanuel, Linda Thumba, Warku Habte Gabul, Kowsar Ahmed, Gebeyehu Asire Mekonnen, Blen Tarekegn, Aristides Cabral Nhanala, Paula S. Kent, Tigistu Adamu Ashengo, Albert W. Wu, Damtew Woldemariam Dagoye, Melanie S. Curless, Sarah Fisseha, Firew Ayalew, Melaku Gebremichael, Bhakti Hansoti

**Affiliations:** ^1^Jhpiego, Addis Ababa, Ethiopia; ^2^Department of Emergency Medicine, Johns Hopkins University School of Medicine, Baltimore, MD, United States; ^3^Armstrong Institute, Johns Hopkins University School of Medicine, Baltimore, MD, United States; ^4^Department of Hospital Epidemiology and Infection Control, Johns Hopkins Hospital, Baltimore, MD, United States

**Keywords:** IPC, safety officer, Ethiopia, COVID-19, infection prevention and control, training of trainers

## Abstract

**Background:**

Effective infection prevention and control (IPC) was central to keeping healthcare workers (HCWs) safe during the COVID-19 pandemic. However, as the pandemic continued, the maintenance of high-quality IPC practices waned, placing HCWs at increased risk of infection. A COVID-19 Safety Officer (SO) program was piloted by the United States Agency for International Development (USAID)-funded Reaching Impact, Saturation and Epidemic Control (RISE) project across two health facilities in Ethiopia, which trained clinical and non-clinical HCWs on IPC protocols to promote safe practices in patient care areas. We sought to evaluate the implementation and effectiveness of the SO program in improving IPC practices within the clinical setting.

**Methods:**

This is a post-implementation evaluation of the SO program, implemented in two hospitals in Ethiopia between May 2022 and December 2022. Participants completed a 4-day course on COVID-19 epidemiology, IPC, safety communication, and learning theory as a part of the Training of Trainers component (*n* = 23), and were posted in clinical wards to provide staff training and support to maintain IPC protocols. The program was evaluated at 6 months using the Reach, Effectiveness, Adoption, Implementation, and Maintenance (RE-AIM) framework. Effectiveness was measured using direct observation of IPC practices across intervention sites. Implementation outcomes were measured using surveys and qualitative interviews to capture training cascade, knowledge, comfort, acceptability, and maintenance.

**Results:**

Participants were able to cascade training to an additional 167 clinical (67.6%) and 80 non-clinical (32.3%) staff across both sites. Direct observation of clinical staff at 6 months showed that 95% (59/62) wore at least a surgical mask with patients and were compliant with masking and/or distancing protocol. Clinical interviews revealed that SOs contributed to increased perceived comfort with screening and isolation procedures and environmental cleaning procedures.

**Conclusion:**

The SO training program was widely adopted, and effective in improving the implementation and comfort of maintaining IPC practices in clinical settings.

## Introduction

The COVID-19 pandemic placed an immense burden on frontline healthcare workers (HCWs) who were tasked to provide care in high-exposure settings. The duration of contact with infectious patients, high patient workload, the potential for spread of the disease to family members, resulting separation from families, and knowledge of infection risk for providers contributed to increased mental stress and physical exhaustion of HCWs (1–3). In Africa, HCWs faced additional challenges due to resource shortages and lack of capacity to care for infectious patients due to limited critical care beds, trained clinical staff, and transportation options (1, 4, 5). Moreover, inadequate training on infection prevention and control (IPC) protocols and gaps in the implementation of hospital safety standards increased the potential for exposure among HCWs (2, 6, 7). In July 2020, the World Health Organization (WHO) reported that HCWs throughout Africa represented 5 to 10% of all COVID-19 infections on the continent. An assessment of hospital capacity for infection prevention conducted by WHO across over 30,000 facilities revealed that only 16% of surveyed facilities scored above 75% for adequate control measures. The major IPC barriers identified included overcrowding, with only 7.8% of facilities having the capacity to triage and isolate infectious patients, compounded by HCW shortages and a lack of staff trained in IPC practices (8).

The Ethiopian Ministry of Health (MOH) has made efforts to standardize practices across the country through the publication of the 2012 Ethiopia National Infection Prevention Guidelines (9, 10). Since then, numerous studies have found provider knowledge on the topic to range between 38.6 to 70% while compliance with prevention practices ranged between 23 to 66% (5, 6, 11, 12). Access to IPC guidelines, training, a positive attitude toward infection prevention practice, and availability of personal protective equipment (PPE) were associated with safe practices and lower healthcare-associated infection among HCWs (6, 11). Notably, HCWs at facilities burdened with high workloads demonstrated lower infection prevention practices (5, 12, 13). The presence of a dedicated IPC committee varied across different hospital settings and served as a positive predictor of HCWs knowledge of IPC (14). Despite high HCW knowledge, the study also demonstrated that interactions with the IPC team were limited, resulting in poorer compliance (14, 15). Studies in Ethiopia have documented low compliance with hand hygiene and other infection prevention practices (16, 17). These findings indicate the need for increased reinforcement of IPC guidelines to promote a safer clinical environment, with a strategy that does not increase the burden of responsibilities on clinical HCWs.

To address this gap, a COVID-19-focused Safety Officer (SO) Program was introduced as a pilot in Ethiopia. The intervention, implemented by the Reaching Impact, Saturation and Epidemic Control (RISE) project funded by the U.S. Agency for International Development (USAID), sought to build the capacity of clinical and non-clinical hospital staff to strengthen IPC practices within health facilities. The program was initially developed in a US-based system (Johns Hopkins Hospital) and adapted to the local context. The adaption included using clinicians as safety officer champions rather than using non-clinicians as was done at the Johns Hopkins Hospital, and using a phase-based approach to training to expand the intervention and ensure sustainability. In addition, training materials, coaching and mentoring, and survey tools were adapted in collaboration with the MOH and implementation hospitals. We hypothesized that the presence of SOs would improve HCW knowledge and adherence to IPC guidelines, thereby reducing the infection risk and fostering a greater sense of safety. This paper describes the implementation of the SO program in Ethiopia and measures the impact of the program on local IPC practices during the pandemic.

## Methods

This is a single time point, post-implementation evaluation study using the Reach, Effectiveness, Adoption, Implementation, and Maintenance (RE-AIM) framework for an intervention to train clinical and non-clinical HCWs on enforcing IPC practices across two health facilities in Ethiopia. The study team first conducted a baseline needs assessment to inform the adaption of the US-based training materials to the local context.

The training component of the intervention occurred in two phases. During phase 1, all participants from the two intervention facilities (*n* = 23) completed a 4-day course on COVID-19 epidemiology, IPC, safety communication, and learning theory as part of the Training of Trainers (TOT) component (June 1–4, 2022). Upon completion of this TOT training, participants were evaluated for knowledge and key competencies and then were designated as “SO Champions.” Phase 2 of the intervention was an expansion of the service into selected units of the two hospitals. Newly trained SO Champions from phase 1 were encouraged to conduct cascade trainings at their home institutions while continuing to work in their current role. SO Champions delivered trainings on COVID-19 epidemiology, IPC, and safety communication to other clinical and non-clinical members (*n* = 243) of their respective institutions.

The trainings were conducted on August 17 and 18, 2022 at Hawassa University Hospital and August 31 and September 1, 2022, at University of Gondar Hospital. During cascade training, SOs were supervised and coached to ensure the quality of the trainings. Upon completion of the phase 2 SO Champion-led cascade training, newly trained participants were also awarded the title of Safety Officer (SO). Organizational and technical support for the cascade trainings was provided by the RISE project to ensure fidelity and standardization of training. Technical support included self and peer assessments among facilitators, and feedback from trainees to improve training quality.

**Figure 1:**
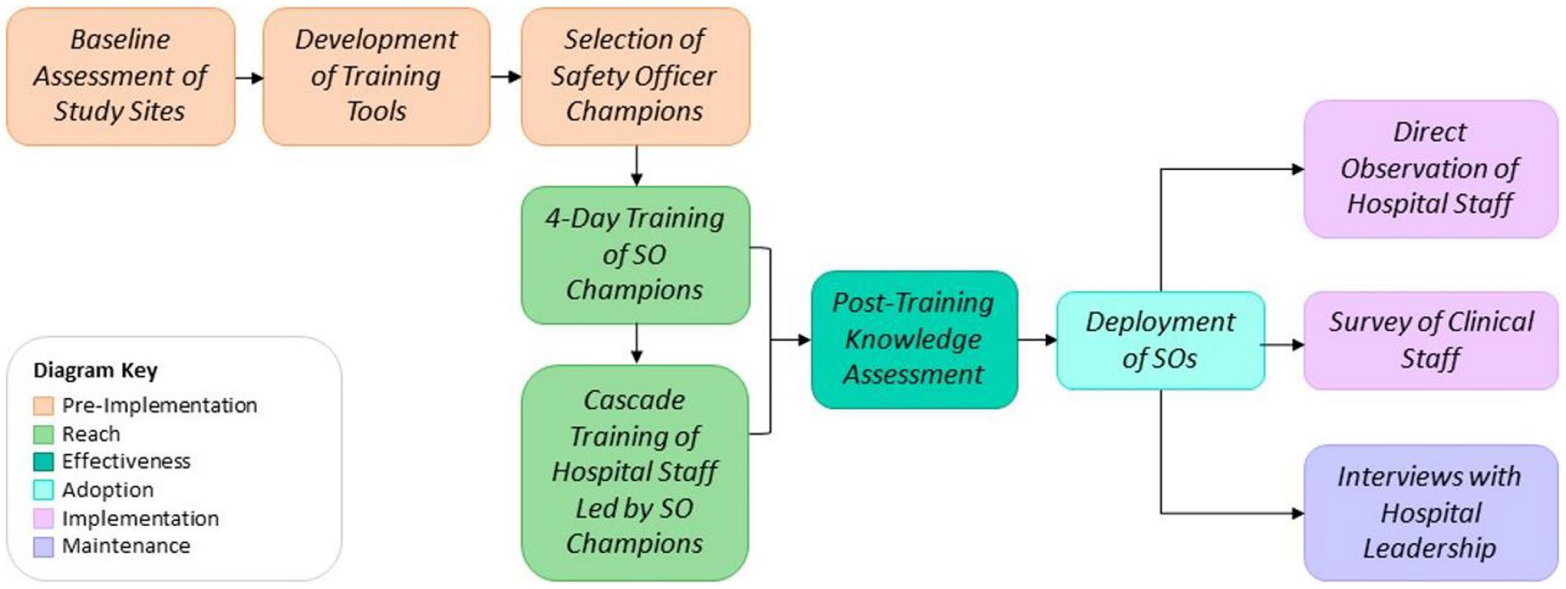
Stepwise overview of safety officer program design implementation and evaluation.

The post-training knowledge evaluations were conducted for participants in both phase 1 and 2, immediately following their respective trainings. Field-based direct observation, surveys, and interviews were completed in parallel at both university hospitals approximately 6 months after the original training intervention from November 3–16, 2022. An overview of the program, as well as the implementation and evaluation strategy, are provided in Figure 1.

### Intervention

The intervention was a SO training program for clinical and non-clinical HCWs to support HCWs in the clinical environment by observing IPC practices and providing real-time feedback to HCW when IPC protocols are not followed (18). Clinical and non-clinical HCWs who completed the training (either in phase 1 or phase 2) were designated as SOs and were responsible for promoting adherence to IPC practices through ensuring resource availability by taking stock and placing orders, addressing knowledge and awareness gaps, and providing real-time interventions in instances of staff non-compliance.

The training incorporated interactive presentations, demonstrations, audio visual teaching materials and hands on training. Along with the theoretical session the participants were expected to demonstrate two major competencies which were hand washing and donning and doffing skills. The initial TOT SO training program (phase 1) had two components: (1) training on COVID-19 epidemiology for clinical and non-clinical staff, IPC knowledge and communication skills to support IPC practices, and (2) training on how to implement the SO training program within a health facility. The program was initially developed and successfully implemented in Baltimore, United States, at the Johns Hopkins Hospital by the Healthcare Epidemiology and Infection Control (JHH-EICH) team in response to HCW needs during the COVID-19 pandemic, and was adapted to the local context for this study.

The JHH-EICH team and the Johns Hopkins Armstrong Institute for Patient Safety and Quality collaborated with the RISE project staff in Ethiopia to adapt the intervention to the local context. The RISE project in collaboration with the Ethiopia Ministry of Health identified the clinical sites for implementation, engaged local stakeholders and facilitated the training and evaluations.

### Safety officer program participant recruitment

Convenience sampling was employed to select study participants from whom data on IPC practices were collected. This sampling method was used to obtain real time practice from clinicians who had completed the training to practice the SO interventions and were on duty during the time of data collection.

During Phase I, participants were selected in communication with the MOH and hospital leadership. The criteria for selection included having good reputation on IPC practices in their department and adequate facilitation skills to train others. During phase 2, participants were selected by the unit heads from the clinical departments to receive the SO training.

### Study setting

The SO program was implemented at two health facilities in Ethiopia, the University of Gondar and Hawassa Comprehensive Specialized Teaching Hospitals. The two hospitals were selected purposively since they are high volume university teaching hospitals and provide an enabling environment including leadership commitment to host the intervention. The University of Gondar Hospital acts as the referral center for four district hospitals in the area. It has a range of specialties, including pediatrics, surgery, gynecology, psychiatry, HIV care, and an outpatient clinic, and serves a population of four million across the region. It has 21 wards and employs approximately 2,100 clinical staff and 1,500 non-clinical staff. Similarly, Hawassa University Teaching Hospital serving the Sidama, Oromia and Southern Nationas and Nationalites regions, boasts a comprehensive suite of sub-specialties, including pediatrics, surgery, gynecology, internal medicine, HIV care, oncology, orthopedics and an outpatient clinic It serves a catchment area of five million and has 15 wards and employs 846 clinical staff and 700 nonclinical staff.

### Evaluation strategy and outcome measures

The primary outcome of this evaluation was the effectiveness of the training program in promoting adherence to IPC protocols, measured through direct observation. Secondary outcomes included HCW knowledge acquisition, as well as behavior change and integration of the intervention by health facility leadership. The overarching evaluation utilized the Reach, Effectiveness, Adoption, Implementation, and Maintenance (RE-AIM) framework. RE-AIM is an evaluation framework, conceptualized over 20 years ago to address the well-documented failures and delays in the translation of scientific evidence into practice and policy (19). RE-AIM was designed to translate research into practice, and can be applied at multiple levels (i.e., individual, organizational and health system), and employs a mixed methods approach to data collection (20). Consideration of the RE-AIM variables ensures the development of effective, sustainable, and evidence-based interventions (18, 21). The evaluation measures across each of the domains is presented in Table 1. Reach was measured using a survey during training. Effectiveness and Adoption were measured using a combination of staff knowledge surveys and direct observation of IPC practices. To assess fidelity, staff were asked to recall if they were comfortable with performing various IPC practices and if they received SO support during the week prior. To assess maintenance, we conducted interviews with key stakeholders and hospital leaders.

**Table 1:** RE-AIM evaluation measures and data collection strategy.

Domain and definition	Method of collection	Specific measures
Reach: The absolute number, proportion, and representativeness of individuals who are willing to participate in intervention/program	Survey	Number and proportion of clinical and non-clinical staff trained by the SO program
		Demographic characteristics of the staff reached (i.e., age, gender, profession)
		Number of clinical staff in intervention sites who recall interacting at least once in the prior week with a safety officer
Effectiveness: The impact of an intervention on important outcomes	Survey	Subjective measure of HCW feeling safe in the clinical environment
	Training evaluation	Changes in knowledge, attitudes, and behavior related to IPC practices
		Staff satisfaction and perceived value of the SO program
	Direct observation	Number of COVID-19 infections/IPC violations
		Safety audit survey observations Wearing approved eye protection Staff wearing masks/N95 Staff social distancing Staff masked with patient Appropriate donning/doffing performed
Adoption: Actualization of intervention within the implementation setting	Direct observation	Number of clinical units that adopted the practice of using safety officers for IPC out of all clinical units that manage COVID patients
Implementation: At the setting level, implementation refers to the intervention agent’s fidelity to the various elements of an intervention’s protocol.	Survey	SO program survey: Were you adequately trained in IPC? At least once a shift, do you encourage staff to wear PPE? Do you spend >70% of time in the clinical area? Do you refill PPE supplies?
Maintenance: The extent to which the intervention becomes institutionalized	Interviews	Survey of hospital leadership and policy changes at 6 months in terms of IPC practices at the institution.

### Data collection and analytical plan

Data were collected using a combination of training logs, pre/post training evaluations for phase 1 and phase 2 participants, direct observations, semi-structured interviews and clinical staff surveys (see Figure 1). In addition to direct observation and surveys, interviews were conducted with hospital leadership to ascertain maintenance. All data was collected by the RISE project technical team. The technical team was oriented to the data collection tools and strategy by the RISE Monitoring, Evaluation, and Learning Advisor to ensure data quality. A simple descriptive analysis was performed using STATA version 17.

### Ethical considerations

Participation in the SO program was voluntary. The study team explained the objectives of the program and informed the staff members of voluntary participation. Verbal consent of participants was obtained prior to the completion of surveys, interviews, and direct observation. Approval for this study was received from Johns Hopkins University Bloomberg School of Public Health institutional Review Board (IRB no 23130, Not Human Subjects Research/ Public Health Practice with PI Bhakti Hansoti and co-PI Firew Ayalew Desta) as well as the Ethiopia Public Health Association Institutional Review Board (IRB reference no EPHA/DG/144/22, Ethical Approval with PI Bhakti Hansoti and co-PI Firew Ayalew Desta).

## Results

In June 2022, 23 clinical HCWs were selected to participate in the TOT training for phase 1 (14 from the University of Gondar Hospital and 9 from Hawassa University Hospital). The participants included environmental health specialists, nurses, midwives, physicians, and clinical managers. The majority of participants were male (78.3%), and the most common profession was nursing (65.2%). Participants from phase 1 were designated as SO Champions, and they were responsible for organizing and leading the Phase 2 cascade trainings. In phase 2, an additional 80 (40 per site) non-clinical HCWs including cleaners, porters, guards, and janitors, and 167 clinical staff, including nurses and midwives, and public health professionals participated in local cascade trainings (Table 2), and were designated as SOs upon completion of the training.

**Table 2:** Demographics of Phase 1 and Phase 2 participants by site.

Demographic category	University of Gondar Hospital [*n* (%)]	Hawassa University Hospital [*n* (%)]
**Study setting**		
Clinical staff	~2,400	846
Non-clinical staff	~1,500	700
Hospital wards	21	15
**Phase 1: Safety officer champion training**		
Clinical staff trained (*n* = 23)	14 (61%)	9 (39%)
Number of wards with safety officers [*n* = 21 Gondar; n-15 Hawassa]	6 (29%)	6 (40%)
**Phase 2: Cascade safety officer training**		
Clinical staff trained (*n* = 186)	89 (53.3%)	78 (46.7%)
Non-clinical staff trained (*n* = 80)	40 (50%)	40 (50%)
Number of wards with safety officers [*n* = 21 Gondar; n-15 Hawassa]	21 (100%)	15 (100%)
**Profession/Qualifications**		
BSc nurse (*n* = 120)	62 (M 60%; F 40%)	58 (M 54%, F 46%)
BSc midwife (*n* = 22)	12 (M 33%, F 67%)	10 (M30%, F 70%)
Public health professionals (*n* = 16)	10 (M 80%, F 20%)	6 (M 67%, F37%)
Environmental health specialist (*n* = 4)	2 (M 100%)	2 (M 100%)
Other (*n* = 5)	3 (M 100%)	2 (M 100%)
Non-clinical (*n* = 80)	40 (M 43%; F 57%)	40 (M 15%; F 85%)

### Effectiveness

Median pre-and post-intervention knowledge tests scores (*n* = 23), increased from 50% [IQR: 41–59] to 77% [IQR: 73–82] (*p* < 0.005). Following the SO Champion Training in Phase 1, 100% of participants were proficient in hand hygiene and PPE donning/doffing. In follow-up direct observation visits, 95% (59 of 62) of clinical staff were observed wearing at least a surgical mask when with patients and were compliant with mask usage or distancing when unmasked. Due to the low volume of COVID-19 cases, *in situ* donning and doffing events were unable to be observed and none of the clinical staff at either hospital wore approved eye protection or N95 respirator masks.

### Reach and adoption

The program successfully trained clinical (68%) and non-clinical staff (32%) with cascade training. However, only 8% of staff surveys in the clinical units recalled being approached by a SO in the week prior to data collection. In contrast to the low recollection of SO support by clinical staff members, follow-up interviews revealed that SOs were present across 12 wards in Phase 1, which expanded to 36 wards in Phase 2.

### Implementation of program

Clinical staff from both facilities (*n* = 62) were surveyed to assess fidelity. Staff most frequently recalled SO support when caring for patients with COVID-19 (43, 69%), receiving hand hygiene training (53, 85%), and with environmental screening procedures (47, 76%) (Table 3). Few staff (*n* = 14, 23%) recalled support with donning and doffing procedures. There were some performance differences between the two facilities on some indicators. For instance, SO support on management of patients with COVID 19 was recalled by a higher percentage of workers at Gondar University hospital (92%) compared to Hawassa University Hospital (38%) (Table 3).

**Table 3:** Survey of clinical staff on SO support recall (*N* = 62).

Indicator	University of Gondar Hospital [*n* (%)] [*N* = 36]	Hawassa University Hospital [n (%)] [*N* = 26]	*n* (%)
Staff recalling SO’s support with management of patients with COVID-19	33 (92%)	10 (38%)	43 (69%)
Staff recalling SO’s support with donning and doffing PPE	1 (3%)	13 (50%)	14 (23%)
Staff recalling SO’s support with Hand Hygiene training	37 (97%)	18 (69%)	53 (85%)
Staff recalling SO’s support with screening and isolation protocols	22 (61%)	4 (15%)	26 (42%)
Staff recalling SO’s support with environmental cleaning procedures	36 (100%)	11 (42%)	47 (71%)

All surveyed staff felt comfortable managing patients with COVID-19, donning and doffing PPE, and screening and isolation procedures regardless of their recall if SOs provided support or not. Only 46 (74%) staff reported feeling comfortable with screening and isolation protocols and only 44 (71%) reported feeling comfortable with environmental cleaning procedures. Comfort was higher in HCWs who recalled SO support compared to those who did not. HCWs who recalled SO support reported higher comfort with screening and isolation protocols (85%, 22/26 vs. 69% 24/36), and higher comfort with environmental cleaning procedures (85%, 40/47 vs. 27%, 4/15).

### Maintenance

Feedback interviews with hospital management revealed a felt need for the SO Program within the health facilities. They also reported improved adherence to IPC practices, and management from both facilities expressed that they intended to maintain the cadre of SOs after the project. Further, the use of continuing education activities enabled the maintenance of competencies and awareness of the support provided by SOs that enhance IPC practices for staff and patient safety.

## Discussion

The COVID-19 SO program intervention in Ethiopia successfully trained clinical and non-clinical HCWs on IPC practices. The pre-test evaluations revealed gaps in knowledge of IPC practices among HCWs chosen to participate in the SO program, particularly in cleaning, hand washing, and donning and doffing PPE, which was unexpected almost 2 years into the COVID-19 pandemic (22). Effective use of PPE has been demonstrated as an effective strategy to mitigate workplace COVID-19 transmissions (23). Maintaining clinical competencies in IPC practices to ensure staff safety requires continuous education and re-training. The lack of baseline knowledge in the SO Program training participants despite ongoing threats of infection acquisition in the workplace reinforces the need for continuing IPC education. Burnout, overwhelming clinical burdens, and complacency may have played a role in the baseline knowledge of IPC practices that we found in our initial evaluation.

Our study demonstrated that engaging SO champions through the TOT program was instrumental in cascading training to the health facilities. Colleagues in the UK developed a similar IPC intervention with a TOT component and highlighted this approach as an effective strategy to rapidly disseminate training during an acute pandemic, but raised caution that there may be challenges in maintaining competencies across the cascade (24). Other studies have shown that a TOT package that combines adult learning theory with interactive practice and teach-back techniques is most likely to improve knowledge acquisition and the likelihood of successful knowledge transfer (25, 26).

Although our findings demonstrated high HCW comfort with hand washing and mask use and lower levels of comfort among clinical staff with donning/doffing, screening, and isolation procedures, we believe that this does not necessarily demonstrate failure of the program. We feel that the findings were confounded by a significant reduction in COVID-19 cases at the time of the evaluation, which limited the encounters requiring PPE and reduced HCW needs for interactions with SOs, leading to lower than expected rates of recall. A study conducted prior to the pandemic revealed lower compliance (74%) for hand washing compared to our study (85%). We also wonder where the poor recall may reflect the hierarchical structure of the healthcare workforce, non-clinical SOs (who are ancillary members of the care team) may have had challenges providing feedback and communication to clinical staff members, thus reducing the number of interactions (27). Further, the commitment from the SO champions at University of Gondar Hospital and facility management team at Hawassa University Hospital may be attributed to an observed performance difference in some IPC practice indicators.

Provider burnout has been reported to be highest in those who continued to treat patients with active COVID-19 illness (28). Other factors shown to contribute to burnout include increased perceived threat of COVID-19, longer working time in quarantine areas, working in a high-risk environment, working in hospitals with inadequate and insufficient material and human resources, increased workload, and lower level of specialized training regarding COVID-19 (29). We hypothesize that, during emergency situations, the presence of additional personnel trained in IPC practices not only increase comfort but also alleviate burnout from healthcare workers.

### Limitations

This is limited study that was conducted to evaluate the implementation of a clinical innovation to improve IPC practice. During the time of our evaluation COVID cases had dropped significantly, which likely impacted observed practices. Furthermore, our observations may be impacted by the significant challenges with limited availability of resources, including PPE and cleaning supplies. Also, while the training was designed to improve IPC practice, we understand that the SOs encompassed a range of cadre, from cleaning staff to nurses, and as such we do not know how much of their role was designed to assist with aiding IPC practices vs. ensuring supply availability. Strategies were adopted, such as onboarding of hospital leaders, to ensure that all health care professionals were aware of the presence of SOs in their venues, but we do not know if the role of SOs was clearly understood by all allied health professionals. Despite these limitations we demonstrate that SOs contributed to considerable improvement to IPC practice adherence in both venues.

## Conclusion

Adherence by HCWs to IPC guidelines plays a critical role in preventing the spread of infections and promoting a safer clinical environment. Despite the presence of an IPC team and existing guidelines, optimal practices are not always observed. The COVID-19 SO Program addressed barriers to IPC by promoting safe practices and cascading the knowledge and skills to both clinical and non-clinical staff. The program was accepted by frontline HCWs and increased their level of comfort with executing IPC protocols. This study demonstrates the benefits of both the TOT methodology and IPC training. The training of non-clinical cadres provides potential leverage to augment support for maintaining high quality IPC practices in clinical venues that provide care for patients with highly transmissible infections. Further study is needed to evaluate knowledge retention, the ideal proportion of staff who should receive this training and opportunities to explore just in time training strategies for rapid scale-up during health emergencies.

## Data Availability

The raw data supporting the conclusions of this article will be made available by the authors, without undue reservation.
